# Morphological and Molecular Changes of the Myocardium After Left  Ventricular Mechanical Support

**DOI:** 10.2174/157340308785160606

**Published:** 2008-08

**Authors:** Hideo A Baba, Jeremias Wohlschlaeger

**Affiliations:** Institute of Pathology and Neuropathology, University Hospital of Essen, University of Duisburg-Essen, Germany

**Keywords:** Congestive heart failure (CHF), ventricular unloading, left ventricular assist device (LVAD), reverse cardiac remodeling, morphology, weaning.

## Abstract

Left ventricular assist devices (LVAD) are currently used to either “bridge” patients with terminal congestive heart failure (CHF) until cardiac transplantation is possible or optionally for patients with contraindications for transplantation (“destination therapy”). Mechanical support is associated with a marked decrease of cardiac dilation and hypertrophy as well as numerous cellular and molecular changes (“reverse cardiac remodeling”), which can be accompanied by improved cardiac function (“bridge to recovery”) in a relatively small subset of patients with heart transplantation no longer necessary even after removal of the device (“weaning”). In the recent past, novel pharmacological strategies have been developed and are combined with mechanical support, which has increased the percentage of patients with improved clinical status and cardiac performance. Gene expression profiles have demonstrated that individuals who recover after LVAD show different gene expression compared to individuals who do not respond to unloading. This methodology holds promise for the future to develop read out frames to identify individuals who can recover after support. Aside from describing the morphological changes associated with “reverse cardiac remodeling”, this review will focus on signal transduction, transcriptional regulation, apoptosis, cell stress proteins, matrix remodeling, inflammatory mediators and aspects of neurohormonal activation in the failing human heart before and after ventricular unloading.

## 1. INTRODUCTION

Chronic heart failure (CHF) is a major cause of morbidity and mortality in both western industrialized and developing nations and causes considerable economic burden to the medical systems [1-3]. Despite medical treatment is continuously being improved, cardiac transplantation is the only curative approach to CHF of various aetiologies. However, the number of donor hearts is limited and has been declining since the last decade of the 20^th^ century [2]. The need for intervention in acute heart failure and the long waiting periods for donor organs consequently led to the development of left ventricular assist devices (LVAD). LVAD are electrically powered either pulsatile or non-pulsatile pumps or turbines, which can be installed extracorporally or intrathoracically parallel to the circulation, i.e. they transport blood from the left ventricle to the ascending aorta and thereby provide profound volume and pressure reduction and restore systemic blood pressure and flow to near normal levels. This leads to a normalization of the neurohormonal and local cytokine milieu contributing to myocardial recovery [4,5]. LVAD are used either as a “bridge to transplantation” or to re-establish basic cardiac function without transplantation (“bridge to recovery”). Optionally, it can be used in patients with contraindications for transplantation (“destination therapy”). Improved survival rates of patients treated with LVAD until cardiac transplantation compared to medical treatments could be demonstrated [4,6]. The structural and molecular changes occurring in the myocardium during CHF (“cardiac remodeling”) have been intensively studied in the past [7,8]. There is now compelling evidence that prolonged near-total unloading of the left ventricle in CHF is associated with numerous morphological and molecular changes in the myocardium (“reverse remodeling”) [9,10], which can be accompanied by functional improvement and decreased cardiac dilation [11]. Despite encouraging data on morphological and molecular changes of the affected heart, clinical cardiac recovery sufficient to allow device removal (“weaning”) is reported to occur only in a small subset of individuals [12-16]. In general, it is agreed on that cellular and molecular improvement is greater than clinical cardiac recovery [17]. However, Birks and co-workers were able to show myocardial recovery in a percentage as high as 75% of their studied patients after combined use of ventricular unloading and an aggressive pharmacological regimen consisting of lisinopril (angiotensin converting enzyme inhibitor), carvedilol (β-receptor antagonist), spironolactone (aldosterone antagonist) and lorsatan (angiotensin II antagonist), which are known to reduce ventricular remodeling. This approach is followed by administration of clenbuterol, a selective β_2_-agonist, helping to prevent cardiomyocyte atrophy in response to long-term unloading [18]. Although LVAD no longer increase the risk of transplantation itself [2], there is considerable perioperative morbidity and mortality with patient survival between 60 and 75%. The major limitations to survival are advanced age, sepsis and hepatic/renal dysfunction as well as device-related problems including HLA antibodies, infection and device failure [19]. Our group reported on a case of hypersensitivity myocarditis, which resolved after ventricular unloading [20]. Mechanical ventricular support does not influence the survival rate after transplantation or the incidence and severity of rejection episodes [21]. In addition, patients supported by LVAD have an increased quality of life (QOL) and functional status compared individuals with medical treatment [6]. The present review mainly focuses on the major morphological and molecular changes that occur in the heart during “reverse cardiac remodeling” by ventricular unloading, including some of the latest results from our group.

## 2. CHRONIC HEART FAILURE: CARDIAC “REMODELING” VERSUS “REVERSE REMODELING” AFTER VENTRICULAR UNLOADING BY LVAD

CHF is a complex disease process with a step-wise progression, eventually leading to terminal pump failure, interstitial fibrosis, cardiac dilation, arrhythmias and cardiac death. Various stimuli can initiate this process, which is combined with compensatory attempts of the body to counteract by several pathobiological mechanisms. After unloading, the deteriorated cardiac function can partially recover, which is accompanied by numerous changes on different cellular and molecular levels and systems including changes in gene expression profiles (“reverse remodeling”) (for review see [10]).

### 2.1. Morphological Changes of the Myocardium During CHF (“Cardiac Remodeling”)

Myocardial remodelling denotes acquired pathological states of the heart resulting in rearrangement of normally existing structures and generally concerns the two components of the cardiovascular system, i.e. the myocardium and the vessels, the structure of both can be altered by unfavourable conditions caused by several noxious stimuli that impose increased biomechanical stress to the cardiomyocytes [22]. In response to an increased workload, individual cardiomyocytes react by adaptive hypertrophic growth, i.e. they increase in cell size, volume and mass, or undergo apoptosis, respectively [23,24]. As a result, there is organ enlargement, cardiac dilation and increased sphericity [22]. Although salutary at the beginning by reducing wall tension, hypertrophy eventually becomes a maladaptive process, leading to chronic heart failure (CHF) and cardiac mortality [23,24]. Dilation is followed by increased ventricular wall stress resulting in decreased coronary blood flow, impaired pump function and diminished cardiac output [25]. Moreover, interstitial fibrosis is observed, further hindering systolic and diastolic cardiac function [26].

### 2.2. Morphological Changes of the Myocardium During CHF After Ventricular Unloading (“Reverse Cardiac Remodeling”)

After unloading, an overall reduction of organ size can be observed radiologically. Echocardiography reveals decreased left ventricular diameter and an increased ejection fraction [27-29]. Among others, we have shown a significant reduction of cardiomyocyte diameters after LVAD). Decreased length and volume were repeatedly described as the morphological correlate of decreased organ size [9,30,31]. Whether unloading is associated with decreased interstitial fibrosis or not is still unresolved. Some authors indicate, that a striking reduction of interstitial connective tissue can be observed after LVAD [32]. Bruckner and co-workers confirmed this finding and further report on a reduction of the total collagen content in 9 out of 18 patients with concomitant improvement of the ejection fraction. The authors suggest that the amount of fibrosis and cardiomyocyte hypertrophy before LVAD support is predictive in terms of improvement of the ejection fraction [33]. In contrast, Taketani and co-workers found increased interstitial fibrosis after LVAD support, and another group observed a slight increase of cardiomyocyte diameters together with increased fibrosis after unloading [34,35]. Our group was unable to show any changes of interstitial fibrosis between before and after unloading [36]. These conflicting results indicate the difficulties in proper assessment of changes of interstitial fibrosis rather than differences between the studied cohorts. As indicated previously we think [10], that the striking differences obtained concerning this parameter by various groups is due to methodological problems. Computerized semiquantitative analysis of Sirius Red stained myocardial specimens yield density data such as sectional profile areas per unit area or three-dimensional quantities per unit volume. Interpretation of such data is difficult, because changes of the structures of interest can not be detected when both the latter and the reference structure show equivalent changes, i.e. when changes of both structures are correlated: this is the so-called reference trap in stereology, meaning that false data are obtained when both the structure measured (amount of fibrosis) and the reference structure (the heart) simultaneously show changes in their volumes. This is important to bear in mind when investigating ratios before and after LVAD, because the reference volume (i.e. the heart) decreases in size due to hypertrophy regression. Thus, one approach to circumvent the reference trap problem in the measurement of interstitial fibrosis in future studies would be to assess the heart volume based on MRI studies and extrapolate the volume fraction of interstitial connective tissue measured on histological sections to the absolute volume of fibrosis [10,37-40].

### 2.3. Molecular Mechanisms Involved in Cardiac “Remodeling” and “Reverse Remodeling”

Numerous molecular systems become progressively altered and/or disturbed during CHF, partly causal to CHF and partly as an after effect of proceeding CHF. Some, but not all of them are regarded as initially compensatory mechanisms that become deleterious as the disease progresses. Almost all molecular systems investigated show changes associated with ventricular unloading and simultaneous drug therapy. Klotz and co-workers showed that angiotensin converting enzyme inhibition during unloading resulted in decreased interstitial collagen content and myocardial stiffness compared to controls, who were only mechanically supported [41]. “Reverse cardiac remodelling” can be viewed as a result of both drug-mediated and mechanical LVAD-mediated effects.

#### 2.3.1. Extracellular Matrix

**Fig. (1):**
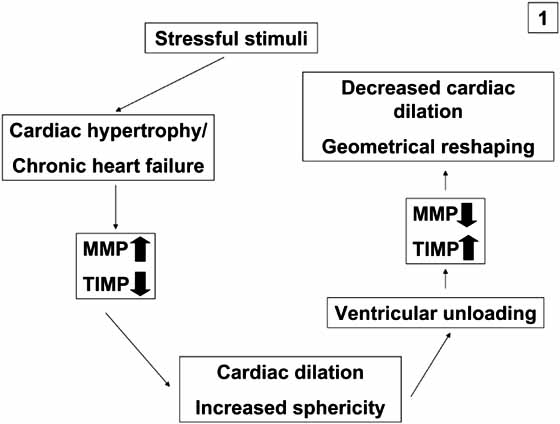
Simplified schematic diagram of the role of matrix metalloproteinases (MMP) and their inhibitors (TIMPS) during chronic heart failure (CHF) and reverse cardiac remodelling: increased expression of MMP and decreased expression of TIMPS during CHF increase turnover of extracellular matrix constituents and favour cardiac dilation, these mechanisms are reversed after ventricular unloading and therefore induce geometrical reshaping of the heart.

The extracellular matrix is necessary for the proper alignment and the coordinated process of transduction of the contractile force generated by the cardiomyocytes. During CHF, severe changes in the extracellular matrix turnover are observed, mediated by members of the matrixmetalloproteinase family (MMP)[42-44]. In summary, MMP are upregulated during CHF, whereas their inhibitors, the TIMPS (tissue inhibitors of matrix metalloproteinases) are downregulated. In particular, MMP3 and MMP9 were found to be increased during CHF, while MMP2 was unaltered. MMP 3 is important because of its ability to degrade a wide range of extracellular matrix (ECM) components including collagen and to activate latent MMP. This altered expression of MMP and TIMPS favours degradation and destabilization of the mechanical scaffold of the heart resulting in ventricular dilation [44]. In contrast, decreased expression of MMP 1 and 9 and upregulation of TIMP 1 and 3 were observed after ventricular unloading followed by a decreased extracellular matrix turnover resulting in less pronounced ventricular remodeling and decreased dilation. Recently it was reported that “reverse remodeling” follows a biphasic pattern. Initially, an increase in Type I and Type III collagen turnover occurs, which is paralleled by a volume increase of the ECM. Subsequently, this turnover decreases as ECM volume decreases, which results in a restoration of the collagen network [45]. The biological mechanism underlying these changes might be that MMP are induced by mechanical stretch of cardiomyocytes. In summary, ventricular unloading reduces wall tension by volume- and pressure reduction and favours the physiological homeostatic balance between MMP and TIMP, thereby facilitating partial geometrical reshaping of the heart [43] (Fig. **1**).

#### 2.3.2. Cardiomyocyte Biology

##### 2.3.2.1. β-Adrenergic Signal Transduction

**Fig. (2):**
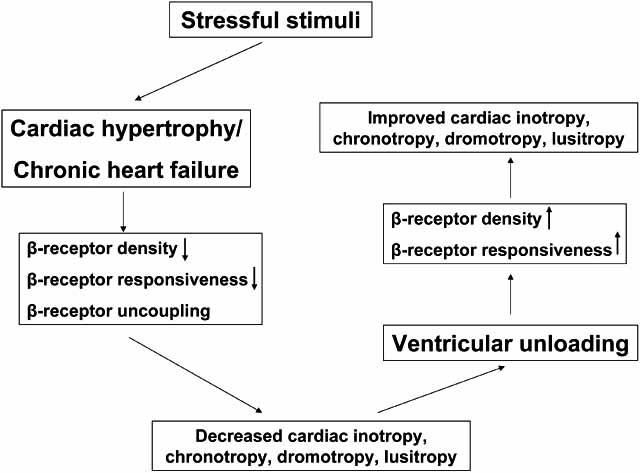
During CHF, besides receptor uncoupling, the density of β-adrenoreceptors and their responsiveness are decreased leading to decreased cardiac function and contractility. Some of these molecular changes are reversed by mechanical ventricular support.

Inotropy, chronotropy, dromotropy and lusitropy are all regulated by β-adrenergic receptors and the subsequent activation of the G protein-adenylylcyclase signal transduction cascade. β-adrenergic receptors are functionally coupled to G proteins and serve as targets of protein kinase A and C as well as β-adrenergic receptor kinases (β-ARK). β-adrenergic receptor signalling can be altered by (1) receptor downregulation due to decreased synthesis or to increased sequestration (long-term regulation requiring hours) and (2) receptor dysfunction as a result of uncoupling from the signal transducing G proteins (G_S_) (short term regulation requiring seconds) and induced by receptor phosphorylation by protein kinases A and C and β-ARK [46]. Isolated cardiomyocytes from failing human hearts show a decreased response to β-adrenergic agonists. The density of β-adrenergic receptors in the myocardium of patients with either dilated cardiomyopathy (DCM) or ischemic cardiomyopathy (ICM) is markedly decreased [47]. After unloading, restoration of β-adrenergic receptor density to near-normal levels is observed. Moreover, response to β-adrenergic stimulation by muscles from unloaded hearts is similar to non-failing hearts. Of note, these changes were not dependent on the duration of ventricular support and receptor density alone did not predict the magnitude of the inotropic response. The mechanism underlying this functional cardiomyocyte improvement may include normalization of the plasma neurohormones and the decrease in both plasma and cardiac cytokine levels after LVAD support [48] (Fig. **2**).

##### 2.3.2.2. Ca^2+^ Homeostasis

Contractile dysfunction during CHF of either aetiology is associated with alterations of Ca^2+^ cycling [49]. Downregulated gene expression of sarcoplasmic endoreticular Ca^2+^ ATPase subtype 2a (SERCA2a), of the sarcoplasmic reticular (SR) ryanodine-sensitive Ca^2+^ release channel (ryanodine receptor [RyR]), and altered regulation of the sarcolemmal Na^+^/ Ca^2+^ exchanger have been reported and appear to correlate with contractile dysfunction [50]. After LVAD support, several authors indicate a recovery of cardiomyocyte contractile strength, coinciding with normalization of magnitude and time course of the Ca^2+^ transient as well as an increased gene expression and protein level of SERCA2a [51,52]. Terracciano and co-workers have demonstrated that clinical recovery from terminal CHF after pharmacological and mechanical support is associated with modifications in excitation-contraction coupling, and sarcoplasmatic Ca^2+^ homeostasis in particular, and not with a reduction of cardiomyocyte cell size. These findings underscore the importance of Ca^2+^ cycling in CHF and its treatment [53].

##### 2.3.2.3. Natriuretic Peptides and Chromogranin A

**Fig. (3):**
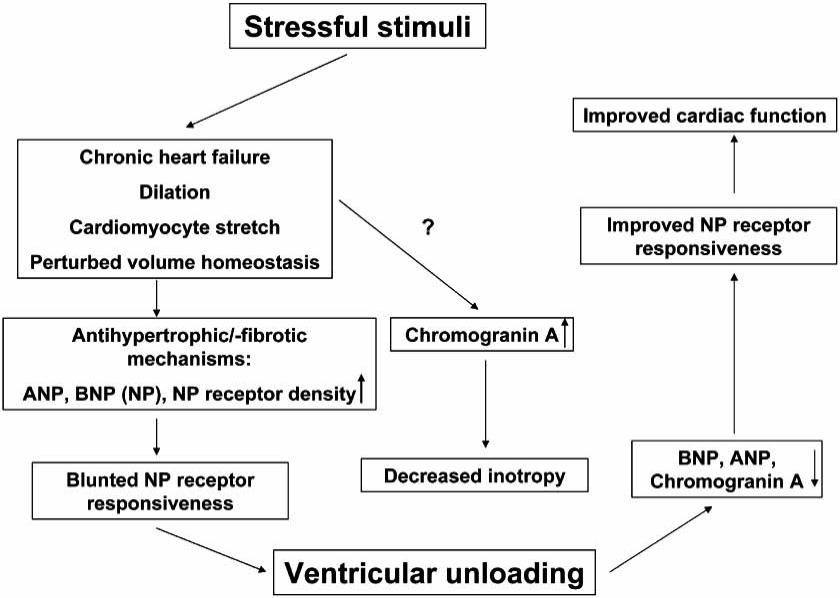
CHF and cardiac hypertrophy are associated with increased expression of ANP and BNP (natriuretic peptides, NP), which are considered to be induced by tensile stretch during cardiac dilation. The NP receptor responsiveness is blunted. Chromogranin A, another neuroendocrine protein is increased during CHF and has been shown to exert cardiodepressive effects in animal studies. LVAD is associated with decreased expression of NP and Chromogranin A.

CHF due to divergent aetiologies is, among other molecular changes, characterized by activation of neurohormonal systems: catecholamines, natriuretic peptides (NP) and components of the renin-angiotensin axis are increased in CHF and were found to have pathophysiological and prognostic implications. Some of these molecules exert local paracrine activation, but their plasma levels were demonstrated to be markers of clinical outcome. Cardiac atrial (ANP) and B-type natriuretic peptides (BNP) are crucial for the maintenance of arterial pressure and volume homeostasis [54-56]. Moreover, they act in an antihypertrophic (ANP) and antifibrotic (BNP) manner *via* guanylyl cyclase A (GC-A) [57]. Both ANP and BNP are elevated in the plasma of CHF patients and these peptide levels are significantly correlated with disease severity. Interestingly, there is a markedly decreased diuresis/natriuresis, vasodilation and vascular synthesis of cGMP in response to exogenous ANP and BNP, indicating a downregulation or impaired receptor or postreceptor responsiveness of GC-A in peripheral tissues from CHF patients. Involvement of enhanced clearance of natriuretic peptides was also suggested [58]. We investigated changes in ANP, BNP and the natriuretic peptide receptor (NPR-C) by quantitative reverse transcriptase polymerase chain reaction (RT-PCR) and determined the activity of the ANP/GC-A system. Increased cardiac ANP and BNP expression in CHF patients is associated with increased expression of the NP metabolizing NPR-C receptors and blunted responsiveness of GC-A to ANP by reduced cGMP synthesis. “Reverse remodeling” after unloading reverses these changes and re-establishes the local responsiveness of GC-A to ANP. Cardiac expression of ANP, BNP and NPR-C mRNA correlated significantly with cardiomyocyte diameters. In contrast to the latter, the levels of the natriuretic peptides are fully reversed to the level of the controls, indicating that their expression is partly independent of cardiac hypertrophy and regulated by CHF associated factors such as cardiomyocyte stretch [59] (Fig. **3**).

**Fig. (4):**
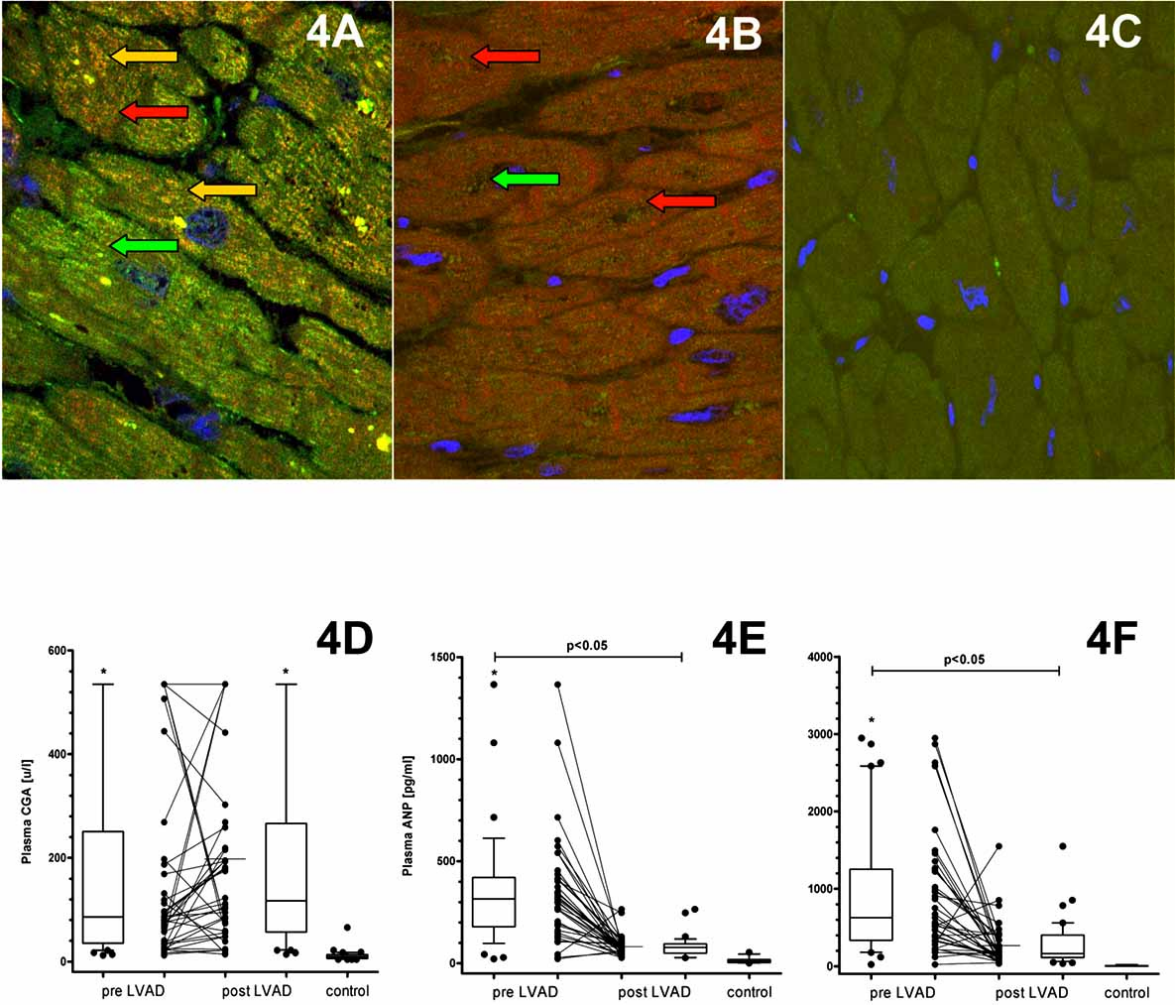
Colocalisation of BNP and Chromogranin A during CHF (A), which is considerably decreased by unloading (B). Normal control hearts are devoid of this colocalisation (C). Red arrow indicates Chromogranin A, green arrow indicates BNP and yellow arrows indicate colocalisation of Chromogranin A with BNP. Despite being significantly decreased in the myocardium after unloading, owing to its low expression in the myocardium, Chromogranin A plasma levels do not reflect hypertrophy regression after LVAD (D). ANP and BNP are significantly decreased in the plasma after unloading (E and F).

Chromogranin A (CGA) is an acidic calcium-binding protein (49 kDa) and is the major soluble constituent in secretory vesicles throughout the neuroendocrine system [60]. CGA was found to be significantly upregulated during CHF and is co-stored with catecholamines and NP. CGA is secreted into the circulation with a long-term plasma half-life of approximately 18 minutes. Thus, CGA constitutes an index of a steady activation of the neuroendocrine system rather than a transient response to stress [61,62], and serves as an independent prognostic marker of CHF severity and mortality in line with ANP and BNP [63]. CGA is a prohormone precursor and is proteolysed by proteases *via* numerous cleavage sites [64] in a tissue-specific manner [65], resulting in the formation of catestatin and vasostatins, which exert a autocrine/paracrine negative feedback control on local catecholamine release [66] and vascular dilation [67]. Vasostatins exert a negative inotropic effect on isolated frog and eel hearts and counteract the actions of β-adrenergic drugs [68,69]. BNP and CGA are co-stored in the myocardium of patients with dilated cardiomyopathy, whereas this co-localisation was not found in healthy controls [70]. We investigated the expression of natriuretic peptides (NP) and CGA by immunohistochemistry and morphometric quantification before and after LVAD. In a different set of patients, CGA was evaluated in the plasma. We demonstrated that in line with ANP and BNP, CGA is significantly increased in CHF compared to healthy controls and decreased by ventricular support. Moreover, sarcoplasmic colocalization of BNP and CGA is diminished after unloading. However, due to its low expression the negative regulation of CGA is not reflected by plasma levels, thus CGA does not appear to be an appropriate biomarker for the monitoring of “reverse cardiac remodeling” after unloading (accepted manuscript) (Fig. **4A-F**).

##### 2.3.2.4. Inflammatory Factors: Cyclooxygenase-2 and Phosphorylated Akt ^(Thr308)^

Patients with CHF are characterized by systemic inflammation, as evident by raised circulating levels of several inflammatory cytokines with increasing levels as the disease progresses. Apart from the myocardium, several other cell and tissue types can contribute to the inflammatory response including leucocytes, platelets, tissue macrophages and endothelial cells. These cells synthesize and secrete inflammatory cytokines such as tumor necrosis factor (TNF) α, interleukin (IL)-1β and IL-6, as well as chemokines [71-75]. Of note, in CHF increased levels of inflammatory mediators do not seem to be accompanied by a corresponding increase in anti-inflammatory cytokines such as IL-10 [72]. Moreover, increased plasma/serum levels of inflammatory mediators are tightly associated with deterioration of functional cardiac performance reflected by diminished left ventricular ejection fraction (LVEF) [72-74]. Importantly, the release of inflammatory mediators from the failing myocardium is not necessarily related to necrotic and/or apoptotic cell death of cardiomyocytes. On the contrary, activation of NF-κB, a major inflammatory mediator in the failing myocardium, has antiapoptotic effects [76]. Mechanical ventricular overload and shear stress, hypoxia, ischemia and oxidized low density lipoprotein cholesterol have been shown to be potent inducers of inflammatory factors from different cell types including cardiomyocytes [77-81]. An interaction between inflammatory response and activation of neurohormonal systems was suggested [82,83]: animal studies and *in vitro* experiments have shown that angiotensin II (ATII) activates circulating leucocytes and promotes adhesion to the endothelium, which in response activates adhesion molecules, chemokines and inflammatory cytokines resulting in the production of reactive oxygen species (ROS) and induction of the transcription factor NF-κB [84]. Similar observations have been made for aldosterone [85,86]. A series of experimental studies have demonstrated that inflammatory cytokines such as TNFα, IL-1 and MCP-1 may directly contribute to the pathogenesis of CHF by inducing contractile dysfunction, ventricular dilation, cardiomyocyte hypertrophy, apoptosis and fibrosis [87-89].

**Fig. (5):**
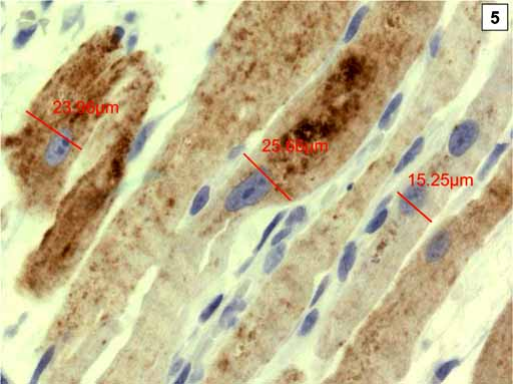
Cardiomyocytes with expression of Cyclooxygenase-2 have significantly larger diameters than COX-2 negative cardiomyocytes.

**Fig. (6):**
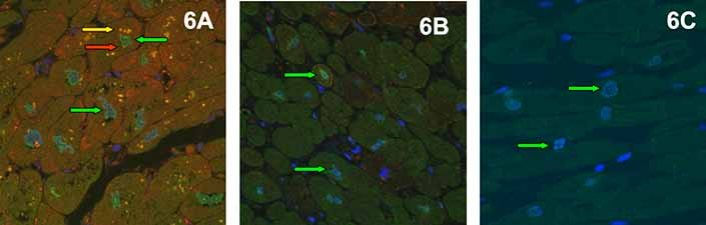
Significant Colocalisation of COX-2 (red signals) and p-Akt ^(Thr308)^ (green signals) resulting in yellow overlay signals (see arrows) during CHF (A). This is reversed by LVAD support (B). Controls do not show signals for COX-2 but only few for p-Akt ^(Thr308)^ (C).

**Fig. (7):**
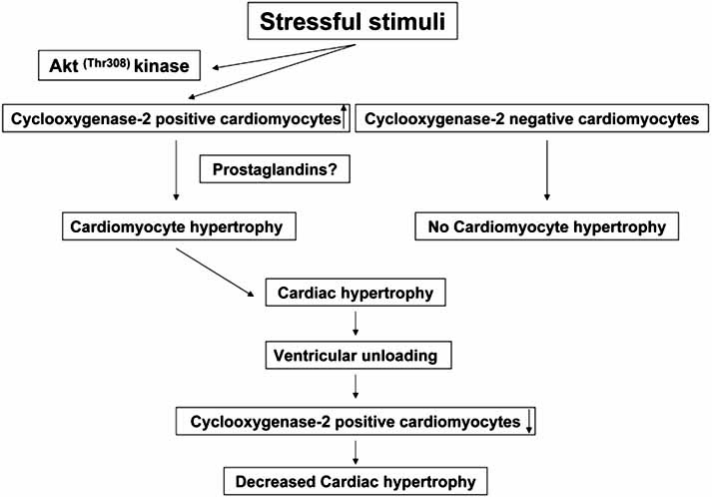
Stressful stimuli lead to COX-2 expression in some, but not all cardiomyocytes, which become hypertrophic leading to organ enlargement. Ventricular unloading significantly decreases COX-2 expression, which is significantly correlated with hypertrophy regression, suggesting a pivotal role of COX-2 in the pathogenesis of maladaptive hypertrophic growth.

Cyclooxygenase-2 (COX-2) is rapidly induced by various cytokines [90] and hypoxia [91], both considered hallmarks of CHF [92]. Consequentially, increased expression of COX-2 has been demonstrated in the failing myocardium [93]. COX-2 expression is regulated, among others [94], through the PI3/Akt pathway, a major player in cell growth and apoptosis [95]. Overexpression of COX-2 was shown to induce Akt phosphorylation at the threonine residue 308 (Thr308), but not at the serine residue 473 (Ser473) *in vitro*, indicating that the COX-2 induced growth stimulus is mediated mainly by phosphoinositide-dependent protein kinase-1 controlled Akt phosphorylation [96]. Moreover, Akt itself was suggested as a promoter of cardiac hypertrophy [97]. We investigated COX-2 and phosphorylated Akt both at (Thr308) and (Ser473) and phosphorylated Erk by immunohistochemistry and quantitative morphometry. COX-2, pAkt ^(Thr308)^, pAkt ^(Ser473)^ and pErk positive cardiomyocytes were significantly increased in CHF compared to normal controls and were significantly decreased by unloading. Moreover, a significant positive correlation between COX-2 and cardiomyocyte diameters as well as pAkt ^(Thr308)^ was noted. In particular, COX-2 positive cardiomyocytes showed significantly larger diameters than COX-2 negative cardiomyocytes, suggesting that COX-2 plays a crucial role in hypertrophic growth during CHF (Fig **5**). Moreover, there was abundant colocalization of COX-2 and pAkt ^(Thr308)^ seen in immunofluorescence doublestaining, suggesting a direct molecular cross-talk between the two proteins, as was suggested by *in vitro* studies (Fig. **6A-C**)[96]. Furthermore, numerous animal studies have shown that hypertrophy and functional deterioration occur upon COX-2 overexpression, and are ameliorated by administration of COX-2 inhibitors, suggesting that COX-2 exerts cardiodepressive effects [98-100]. In summary, we demonstrated a negative regulation of COX-2 after ventricular unloading and suggest a central role for COX-2 in cardiomyocyte hypertrophy, since only COX-2 positive cardiomyocytes showed significant hypertrophy regression after LVAD (Fig. **7**). Moreover, this might be one of the interfaces between neurohormonal activation, systemic inflammation and maladaptive cardiac hypertrophy [101].

##### 2.3.2.5. Signal Transduction and NF-κB

###### 2.3.2.5.1. Transduction Pathways

Among others, three major signal transduction pathways have been shown to be involved in the pathogenesis of cardiac hypertrophy and “remodeling”: (1) the mitogen-activated protein kinases (MAPK) with the extracellular signal-related kinases (Erks), c-jun N-terminal protein kinases (JNKs) and p38 MAPK subfamilies [102]; (2) the Ca^2+^/Calmodulin activated protein kinase (CaM kinase) and phosphatase (calcineurin) [103], and (3) the protein kinase B/Akt and its downstream target glycogen synthase kinase 3β (GSK3β) [104]. All three pathways become activated during CHF in humans. Whereas the Erks are activated by hypertrophic stimuli such as phenylephrine, angiotensin II (ATII) and endothelin-1 [102], the JNK and p38 MAPK are activated by cellular stress and seem to be involved in apoptotic cell death [105]. Apart from acting as an anti-apoptotic factor, Akt is a major repressor of cardiac hypertrophy by inhibitory phosphorylation of GSK3β [106]. The phosphatidylinositol-3-OH kinase (PI3K) is a signalling system that acts through Akt and p70S6 kinase, which is a key factor in angiotensin II (ATII) receptor type 2 mediated cardiac hypertrophy [107]. We investigated the activity of the mitogen-activated protein kinases (MEKs), Erks, Akt, GSK3β, p70S6 kinase, JNKs and p38 in terminal CHF before and after unloading. Western blot analysis revealed a dramatic decrease in dually phosphorylated active forms of Erk-1 and Erk-2 after mechanical support. Also the erk-activating kinases (MEK-1/2) were shown to be significantly less phosphorylated after LVAD. After unloading, Akt is inactivated, whereas GSK3β becomes activated. Besides Akt, another kinase involved in the PI3K/Akt pathway, p70S6 kinase showed a dramatic decrease in its phosphorylation in a subset of patients. Despite p70S6 kinase and its isoform p85S6 are associated with cardiomyocyte hypertrophy mediated by angiotensin II (ATII) receptor type 2, there was no correlation between cardiomyocyte diameter reduction and phosphorylation of p70S6 kinase. In contrast, neither the JNK nor the the p38-mediated signalling cascades were altered under LVAD support in this study, suggesting specific regulation of kinase signalling after mechanical support in humans **in vivo**. In summary, our findings underscore the emerging evidence of MEK/Erks and Akt/GSK3β in the pathogenesis and regulation of cardiac hypertrophy. The inactivation of MEK/Erks and the activation of GSK3β after LVAD are in line with the opposing effects of these two signalling cascades with regard to cardiac “reverse remodeling” [108].

###### 2.3.2.5.2. Transcription Factor NF-κB

NF-κB is a crucial regulator of genes involved in anti-apoptosis and cellular responses to stressful factors [109]. Consequently, upregulation of NF-κB was demonstrated in the failing myocardium [93]. We investigated the immunohistochemical expression of the active p65 subunit of NF-κB before and after unloading and found a significant about twofold decrease upon unloading. Moreover, gel-shift assays were performed to test whether the immunoreactivity for active NF-κB correlates with DNA binding activity, which was significantly decreased after unloading. In summary, we identified abundant NF-κB activity in cardiomyocytes during CHF, which is drastically reduced by mechanical support. Therefore, NF-κB appears to be to be a specific and reverse functional response to molecular signals induced by pressure- and volume overload. Since NF-κB regulates the expression of genes encoding for TNFα, IL-6 and heme-oxygenase, which are increased in CHF and decreased after LVAD, NF-κB may regulate a subset of genes crucially involved in “reverse remodeling” [110].

#### 2.3.3. Apoptosis

Apoptosis is believed to contribute to cardiomyocyte dropout and progressive decline in left ventricular function during CHF [111]. Several different apoptotic mechanisms are at work in CHF and are shortly reviewed in the following paragraph.

##### 2.3.3.1. (Extrinsic) Death Receptor Pathway

*Fas/Fas-ligand pathway. *There is evidence that the Fas/FasL system participates in various types of stress-induced apoptosis in the heart, where cellular stressful stimuli sensitize cardiomyocytes to Fas *in vitro *[112]. Elevated levels of soluble Fas ligand and Fas mRNA has been demonstrated in the failing myocardium [113]. The myocardial Fas system (APO-1/CD95) was shown to respond very sensitive and promptly to ventricular pressure- and volume changes [114]. After unloading, the expression of the anti-apoptotic protein FasExo6Del is decreased, hence decreased cardiomyocyte susceptibility to apoptosis can be assumed [115].

###### 2.3.3.1.1. TNF-Receptor Pathway

Cardiomyocytes can undergo apoptosis after stimulation by TNFα, which is increased in the myocardium during CHF [116]. However, a significant decrease of TNFα in the myocardium of LVAD supported patients was demonstrated [117]. In this respect it must be mentioned, that the role of TNFα in the heart is not entirely clear. It has been shown to be beneficial in an animal model when administered as a pretreatment of ischemia/reperfusion [118]. Further studies are needed to definitively clear the role of TNFα in CHF.

###### 2.3.3.1.2. (Intrinsic) Mitochondrial Pathway

This apoptotic pathway plays a crucial role in CHF. It was shown that release of cytochrome c from the mitochondria and activation of caspase 3 occurs in CHF patients [111].

###### 2.3.3.1.3. Bcl-2 Family

These proteins show either pro-apoptotic (Bcl-2 and Bcl-x_L_) or anti-apoptotic properties (Bax, Bad, Bid, Bnip3), primarily acting *via* the mitochondrial pathway. Upon hypoxia or mechanical stretch, both kinds of Bcl-2 family proteins were found to be induced during terminal CHF [119,120].

###### 2.3.3.1.4. Apoptosis and Ventricular Unloading

Long-term unloading is associated with changes of gene expression involved in the regulation of apoptosis. Upregulation of Bcl-x_L_ was demonstrated after LVAD, suggesting that susceptibility to apoptosis is decreased upon unloading [115]. Also data from our group support the notion of reduced apoptotic index after mechanical support [108].

#### 2.3.4. Cell Stress Associated Factors: Heme Oxygenase-1 and Metallothionein

##### 2.3.4.1. Heme Oxygenase-1

Heme oxygenase-1 (HO-1, HSP 32) is a member of family of heat shock (cell stress) proteins induced by noxious stimuli. HO-1 was demonstrated to act cell-protective and anti-apoptotically. Hence, increased expression of HO-1 indicates profound cellular stress [121]. In CHF, HO-1 is significantly increased in cardiomyocytes and to a lesser extent in arterial smooth muscle cells, endothelial cells and inflammatory cells. After unloading, the HO-1 expression recedes to the level of control hearts. Moreover, in the same study increased HO-1 expression in cultured rat cardiomyocytes exposed to hypoxia and declined to normal levels during normoxic conditions [122].

##### 2.3.4.2. Metallothionein

Metallothionein (MT) is a cell stress protein involved in the inactivation of free reactive oxygen species (ROS) [123]. MT expression was in line with HO-1 found to be increased in CHF compared to controls and significantly decreased after unloading. Decreased numbers of MT-1 positive cardiomyocytes were mainly found in subendocardial areas indicating (reversible) hypoxic damage, further underscoring the notion that unloading improves the myocardial oxygen supply [31].

#### 2.3.5. Gene Profiling Changes after Ventricular Unloading

Some studies investigated differences of gene expression profile in both CHF and after unloading. With regard to the fact, that morphological and molecular changes are clearly more evident than clinical recovery, read-out frames for patients who can be expected to recover sufficiently for device removal versus patients who have fewer changes to be weaned from the device are needed. Furthermore, these investigations might be the basis for future therapeutic strategies and concepts. One study investigated the effect of unloading on myocardial gene expression. Profiling gene expression was compared before and after LVAD support in seven patients with idiopathic dilated cardiomyopathy and end-stage heart failure. A total of 130 gene transcripts achieved the strict criteria for upregulation and 49 gene transcripts for downregulation after LVAD support. In line with the proteomic findings, upregulated genes included a large proportion of transcription factors, genes related to cell growth/apoptosis/DNA repair, cell structure proteins, metabolism, and cell signaling/communication. Hall and co-workers performed statistical analysis of a gene expression library of 19 paired human myocardial samples and found a set of 22 genes that were downregulated and 85 genes that were upregulated in response to mechanical unloading. The analysis revealed a high percentage of genes involved in the regulation of vascular networks including neuropilin-1 (a VEGF receptor), FGF9, Sprouty1, stromal-derived factor 1, and endomucin. Taken together these findings suggest that mechanical unloading alters the regulation of vascular organization and migration in the heart. Moreover, GATA-4 binding protein, a critical mediator of myocyte hypertrophy, was significantly downregulated following mechanical unloading. In summary, these findings may have important implications for defining the role of mechanical stretch and load on autocrine/paracrine signals directing vascular organization in the failing human heart and the role of GATA-4 in orchestrating reverse myocardial remodeling [124]. Another important finding concerning gene profiling changes encoding for cytoskeletal proteins selectively for recovery versus non-recovery was reported by Birks and co-workers: they investigated the gene expression encoding for sarcomeric and non-sarcomeric proteins before and after LVAD and found that recovery is associated with specific changes in gene expression. In particular, an increased gene expression of the nuclear protein Lamin A/C was found in recovery, but not in non-recovery. Interestingly, Lamin A/C gene mutations have been found in families with dilated cardiomyopathy. Despite providing structural integrity of the nuclear membrane, Lamin A/C interacts with intermediate filaments of the sarcomere, the actin-based cytoskeleton and the sarcolemma [37], suggesting recovery of that system after unloading. Moreover, changes in six genes for dystrophin-like proteins occurred. Dystrophin is thought to provide structural support for the cardiomyocytes and mutations can result in cardiomyopathy. Disruption of Dystrophin was demonstrated in CHF, which reversed upon unloading [38,39]. The most striking finding of this study, however, were changes in β-integrin signalling. Integrins are transmembrane receptors linking the extracellular matrix to the cytoskeleton. They are expressed on numerous cell types in the cardiovascular system and are involved in gene expression, cell migration, cell proliferation, differentiation and cell death. Moreover, they have been shown to have function in mechanotransduction (cellular mechanism by which a mechanical stimulus is converted in a chemical signal) in response to physiological and pathophysiological signals [125-127]. Integrins bind directly to components of the cytoskeleton through their cytoplasmic tails and can orchestrate changes in cellular architecture. However, an important limitation of this study is, that the recovered group of patients were treated with clenbuterol, whereas the non-recovered group was not and the effect of the drug on the cytoskleleton remains unclear; however, a small number of clenbuterol-treated non-recovered patients showed changes similar to other untreated patients. In conclusion, this study documented a specific pattern of changes in gene expression encoding for both sarcomeric and non-sarcomeric proteins in patients who recovered and who did not, suggesting that the cytoskeleton plays an important role in both “reverse remodeling” and clinical myocardial recovery [40]. The results yielded by this kind of methodology might serve as one possible read-out frame for patients who can be weaned from the device without subsequent transplantation. Moreover, specific gene expression profiles allow for the differentiation of ischemic versus non-ischemic cardiomyopathy [128].

## 3. CONCLUSIONS AND FUTURE PERSPECTIVES

**Fig. (8):**
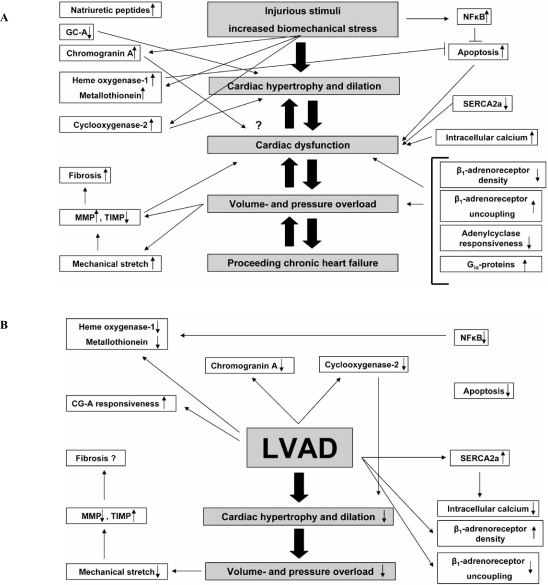
Schematic overview of molecular and morphological changes during CHF (A) and after LVAD support (B).

Numerous noxious stimuli such as chronic ischemia, inflammation or genetic alterations can affect the myocardium and induce rather unspecific compensatory and adaptive changes including cardiomyocyte hypertrophy. Albeit salutary at the beginning these adaptive mechanisms may become maladaptive and deleterious over time and eventually lead to impaired cardiac function. The increased cardiac wall stress and local ischemia may be mechanisms that activate numerous molecular and cellular responses. Protective mechanisms are overrun and the myocardium cannot further adapt to increased biomechanical stress. Neurohormonal activation, inflammatory mediators, alterations in β-adrenergic signal transduction and Ca^2+ ^metabolism and interstitial fibrosis further impair cardiac function (Fig. **8A**). Despite ever improving medical strategies, cardiac transplantation remains the only curative therapeutic approach until now. Due to the shortage of suitable donor organs, LVAD are currently used to maintain cardiac in patients with terminal CHF until a donor organ is available or optionally for patients with contraindications for transplantation as a permanent therapy. As outlined above, the use of unloading by LVAD is associated with changes on the gross anatomical, histological, cellular, molecular and genetic level (Fig **8B**). Although these results are encouraging, there is more or less consent that the clinical cardiac improvement and performance are less pronounced in the majority of cases and only a small subset of patients can be weaned from the device and live without transplantation. The approach described by Birks and co-workers consisting of combined use of mechanical support and medical treatment including clenbuterol holds promising results for the future, as the percentage of patients who can be weaned is reported to be considerably higher. Another important problem in this field is the absence of a suitable serum/plasma biomarker that accurately indicates cardiac recovery under LVAD therapy and could possibly predict the clinical outcome and the changes for successful weaning from the device. ANP and BNP levels are widely used, but these molecules are mainly stretch-induced and levels decrease under support because of volume and pressure reduction, but not necessarily indicate “reverse remodeling” or clinical recovery. We could not show Chromogranin A serum levels to be usable biomarker for this group of patients. Gene expression profiles as performed by Birks *et al*. might serve as a possible read out frame for the future helping to identify patients who can be weaned from the device. However, the master molecular switches orchestrating the process of “reverse remodeling” are still unknown. The heterogeneity of patients concerning the underlying aetiology of CHF, the duration of support, the type of LVAD implanted and medication causes further difficulties, although we never found these parameters to be influential on the parameters investigated. In conclusion, despite the limited clinical improvement occurring only in a subset of patients, a better understanding of the underlying biological mechanisms of cardiac “reverse remodeling” is crucial for the development of future therapeutic strategies in this still intriguing scientific field.
